# Rasagiline Withdrawal Syndrome in Parkinson’s Disease

**DOI:** 10.3390/brainsci12020219

**Published:** 2022-02-05

**Authors:** Paolo Solla, Tommaso Ercoli, Carla Masala, Gianni Orofino, Laura Fadda, Davide Giacomo Corda, Ignazio Roberto Zarbo, Mario Meloni, Elia Sechi, Caterina Francesca Bagella, Giovanni Defazio

**Affiliations:** 1 Unit of Neurology, Department of Medical, Surgical and Experimental Sciences, University of Sassari, 07100 Sassari, Italy; davidegiacomo.corda@aousassari.it (D.G.C.); ignazio.zarbo@aousassari.it (I.R.Z.); eliasechi87@gmail.com (E.S.); caterina.bagella@tin.it (C.F.B.); 2 Department of Medical Sciences and Public Health, Institute of Neurology, University of Cagliari, SS 554 km 4.500, 09042 Cagliari, Italy; gianni.orofino@tin.it (G.O.); fadda_laura@yahoo.it (L.F.); giovanni.defazio@unica.it (G.D.); 3 Department of Biomedical Sciences, University of Cagliari, SP 8 Cittadella Universitaria, 09042 Monserrato, Italy; cmasala@unica.it; 4 IRCCS Fondazione Don Carlo Gnocchi ONLUS, 20148 Milan, Italy; mmeloni@dongnocchi.it

**Keywords:** rasagiline, DAWS, Parkinson’s disease

## Abstract

Parkinson’s disease (PD) patients using dopamine agonists can develop withdrawal symptoms, referred to as dopamine agonist withdrawal syndrome (DAWS), under dose tapering or discontinuation of these drugs. DAWS includes a severe stereotypical cluster of psychiatric and psychological symptoms encompassing severe mood and anxiety disturbances, autonomic symptoms, as well as generalized pain and drug cravings. However, symptoms of withdrawal of dopamine replacement therapies (DRT) are not simply limited to dopamine agonists tapering, as observed in PD patients on deep brain stimulation after dopaminergic drugs withdrawal related to surgery. To date, no DRT-related withdrawal syndrome has been described in PD patients who discontinue rasagiline, an irreversible inhibitor of monoamine oxidase-B (MAO-B). Here we report three PD patients who developed a severe withdrawal syndrome after rasagiline suspension. The syndrome was mainly characterized by prominent psychiatric disorders (depression, anxiety with panic attacks, dysphoria, and agitation) associated with fatigue, generalized pain, and autonomic manifestations (closely resembling symptoms of DAWS). In our opinion, this report suggests the importance of closely monitoring PD patients undergoing rasagiline suspension for withdrawal symptoms and provides interesting points of reflection on the role of rasagiline and other MAO-B inhibitors in mood disorders.

## 1. Introduction

Parkinson’s disease (PD) patients using dopamine agonists can develop symptoms similar to addictive drug withdrawal. This is referred to as dopamine agonist withdrawal syndrome (DAWS) when they necessitate dose tapering or discontinuation of these drugs [1,2]. DAWS includes a severe stereotypical cluster of psychiatric and psychological symptoms causing clinically significant distress or social/occupational dysfunction, closely and temporally related to dopamine agonists tapering in a dose-dependent manner and resistant to levodopa and other psychotropic medications [2,3]. These symptoms encompass affective disturbances such as anxiety, panic attacks, depression, dysphoria, agitation, and irritability, as well as autonomic symptoms such as diaphoresis, flushing, nausea, emesis, and orthostasis, along with generalized pain and drug cravings [2,3].

The causes of dopamine agonist discontinuation have been mainly related to significant side effects, such as hallucinations, cognitive changes, and impulse control disorders (ICD) [4]. The rapid withdrawal of dopamine agonists as a consequence of the introduction of levodopa-carbidopa intestinal gel infusion (LCIG) has been also described [5]. However, symptoms of withdrawal of dopamine replacement therapies (DRT) are not simply limited to dopamine agonists tapering. Indeed, a DRT-related withdrawal syndrome has also been observed in PD patients with deep brain stimulation (DBS) who developed depression and apathy after the abrupt reduction of dopaminergic drugs due to DBS implantation [6].

To date, DRT-related withdrawal syndrome has never been described in PD patients after the discontinuation of rasagiline. Rasagiline is a strong, selective, and irreversible inhibitor of monoamine oxidase type-B (MAO-B) which is used as monotherapy or as add-on to levodopa in PD patients for its effects on prolonging the duration of action of both endogenously and exogenously derived dopamine [7]. For these reasons, rasagiline is considered a well-tolerated and effective drug both in the treatment of early PD [8] and in addition to dopaminergic treatment in PD patients with motor fluctuations [9].

Here we report three PD patients who developed a severe withdrawal syndrome after rasagiline suspension.

## 2. Case Reports

### 2.1. Case 1

A 75-year-old woman with a 6-year history of PD went to our center. Her clinical history was positive for anxiety, constipation, and allergic asthma. She was taking occasionally alprazolam (2 mg/daily) and no other drugs except for antiparkinsonian treatments.

At PD onset, she presented mainly with bilateral rest tremor of the hands, predominant to the left side, without marked signs of bradykinesia and rigidity. No significant cognitive dysfunction was identified. Brain MRI was substantially normal. ^123^I-FP-CIT SCAN evidenced a mild bilateral putaminal uptake reduction. Hoehn and Yahr stage was II, and UPDRS-III score was 8. She was put on treatment with rasagiline 1 mg/daily with mild improvement of tremor after two months (UPDRS-III score 5). Tremor symptomatology progressed slowly, and she did not require any other dopaminergic drugs for the following five years. At 74-years-old, she started to complain of unexplained cutaneous allergic reactions and hypertension. Cardiologic and dermatological consultations did not detect any relevant pathologies. Neurological examination showed bilateral rest tremor, predominant to the left side, without significant rigidity or bradykinesia (the UPDRS-III score was 9). In according with reiterated patient’s requests, who was convinced of a possible allergic effect of rasagiline, the administration of this drug was interrupted.

However, after 10 days from rasagiline withdrawal, she started to present severe affective symptoms, such as marked depression associated with anxiety, dysphoria, and agitation. She also complained of autonomic symptoms, such as nausea and vomiting, as well as fatigue and generalized pain. Neurological examination showed enhanced resting tremor, hypomimia, bilateral bradykinesia, rigidity, and associated postural instability (UPDRS-III score equal to 39, Hoehn and Yahr stage was 3). This disabling impairment required the prompt introduction of levodopa at 300 mg/daily. One month after the introduction of levodopa, although the PD motor symptomatology was clearly improved (UPDRS-III score equal to 22), the affective disturbances were always persistent and severe. Indeed, she complained that levodopa treatment did not alleviate her symptoms since she was unable to perform any kind of daily activity, with repeated requests of increasing her DRT. Severe affective symptoms were present for other six months with the need to further increase dopaminergic treatment, with following spontaneous amelioration.

### 2.2. Case 2

A 68-years-old, a right-handed female patient with an 8-year PD history started with predominantly right-sided rest tremor, bradykinesia, and rigidity. She has been taking rasagiline 1 mg/day since PD onset and rotigotine transdermal patch 4 mg/day for 30 months. Her baseline UPDRS motor score (on state) was 25. Due to an unexplained disabling headache associated with hypertension, she was advised to suspend rasagiline by her treating physician. After rasagiline suspension, she started to complain of severe anxiety, depression, agitation, dysphoria, fatigue, insomnia, and generalized pain. She also experienced panic attacks characterized by shortness of breath, palpitations, and hyperventilation. A significant motor impairment with worsening of tremor and bradykinesia was also reported. For this reason, benzodiazepines were added and rotigotine transdermal patch dosage was increased. Although motor impairment clearly improved, these interventions did not significantly modify psychiatric and other non-motor symptoms, which severely impacted her quality of life, causing severe familiar and social disturbances. At the next follow-up visit, we decided to reintroduce rasagiline at the previous dosage and this measure produced a rapid and dramatic improvement in her non-motor symptoms, with the normalization of the affective and sleep disturbance, without evidence of adverse effects.

### 2.3. Case 3

A 43-year-old male was diagnosed with PD based on right-sided rigidity and bradykinesia. He had no history of psychiatric disease, gambling, or alcoholism. He was started on cabergoline after his diagnosis, but due to burning and tingling in both legs, cabergoline was discontinued and pramipexole was introduced. When he was 47-years-old, the severity of motor impairment increased and levodopa/carbidopa was gradually introduced until the dose of 100/25 mg for three times per day was reached. At 48-years-old, the patient experienced significant motor fluctuations and levodopa/carbidopa was progressively increased over the next two years to 100/25 mg five times per day. At the age of 49, rasagiline 1 mg/daily and melevodopa hydrochloride plus carbidopa two times per day were introduced as adjunctive treatment. In the following 10 years, due to progressive impairment of motor fluctuations, levodopa/carbidopa was progressively increased to 100/25 mg nine times per day. At 61-years-old, due to evidence of REM sleep behavior disorders and insomnia, clonazepam and trazodone were introduced, without significant improvement of sleep disturbances. Thus, his personal senior consultant neurologist decided to discontinue rasagiline administration.

After rasagiline withdrawal, the patient experienced severe anxiety associated with marked dysphoria and depression with episodic marked agitation and aggressivity, lasting for several months. He also complained of fatigue, generalized pain, rigidity, and insomnia. Due to personal problems, the patient did not attend his personal senior consultant neurologist but decided to contact a physician expert on complementary and alternative medicine. Two oil-diluted medical cannabis extracts (Bediol and Bedica) were prescribed, with a progressive amelioration of anxiety and other affective disorders. At 62-years-old, he came to our attention referring that these measures were successful in reducing psychiatric symptoms and explaining that he was experiencing a satisfying medical condition, though mild motor fluctuations were present. Indeed, the patient reported only sporadic and transient episodes of mild anxiety. UPDRS motor score (on state) was 28, while modified Hoehn and Yahr stage was 2.5. Due to his medical condition, we decided to do not reintroduce rasagiline, confirming his pharmacological treatment.

The cases were reported to their country’s pharmacovigilance system, according to the guidelines for submitting adverse event reports for publication [10]. Written informed consent signed by the patients in accordance with the declaration of Helsinki was obtained. Complete information of these three cases has been summarized in Table 1.

## 3. Discussion

To our knowledge this is the first report describing a withdrawal syndrome clearly related to the suspension of a MAO-B inhibitor drug such as rasagiline in three PD patients. Within this context, a recent review did not find any implications of tapering off rasagiline in PD patients, confirming the novelty of our findings in the literature [11]. The withdrawal symptoms that we have described in this study were highly stereotyped and mainly characterized by prominent psychiatric disorders (i.e., depression, anxiety with panic attacks, dysphoria, and agitation). However, other motor and non-motor disturbances were concomitant and, among the latter, fatigue, generalized pain as well autonomic manifestations were the most disabling symptoms. In these three cases, the symptomatology was clearly temporally correlated with rasagiline suspension and, in the patient eligible for the reintroduction of rasagiline, the affective disorder was rapidly and selectively remitted by rasagiline replacement, confirming the drug-specific withdrawal syndrome.

These clinical manifestations of withdrawal in PD patients due to rasagiline suspension closely resembled other DRT withdrawal syndromes, especially DAWS [2]. DAWS is a disabling complication related to DA tapering which encompasses a severe stereotypical cluster of psychiatric and psychological symptoms, such as anxiety, agitation, depression and drug craving, causing clinically significant distress or social/occupational dysfunction, very similar to those observed in patients with rasagiline withdrawal, and strongly linked with ICDs [2,3]. In a subset of patients, DAWS may be an unremitting condition whether therapy with dopamine agonists is maintained or not (even after several years) [12]. Previous studies have suggested that the major determinant of the development of DAWS may be the individual tendency to develop ICD in response to exposure to any DA, rather than other treatment or demographic factors [2,3]. On this basis, it has been suggested that the increased vulnerability to DAWS and ICDs in parkinsonian patients belong to a ‘mesocorticolimbic variant’ of PD, with disproportionate mesocorticolimbic versus nigrostriatal dopaminergic dysfunction [2,13]. However, emerging observations indicate that ICDs should not be considered an absolute requirement, but rather a significant risk factor for DAWS development in PD patients undergoing DA withdrawal [4,14]. In fact, we previously observed the occurrence of DAWS symptoms after DA withdrawal in advanced PD patients, without previous ICD, treated with levodopa-carbidopa intestinal gel infusion [5]. These considerations are in line with other cases of DAWS observed after PD surgery for DBS implantation [6].

An interesting issue regards the effective role of rasagiline suspension in the development of this specific withdrawal syndrome. Indeed, rasagiline is a well-tolerated and effective drug both used in monotherapy in the treatment of early PD [8] and added to levodopa treatment in motor fluctuations [9]. Long-term motor efficacy of rasagiline in early PD has been extensively studied, showing that after 5.4 years only 25% of patients treated with this MAO-B inhibitor progressed to Hoehn and Yahr stage III [15]. Therefore, these data confirm the efficacy of rasagiline in the management of motor symptoms related to nigrostriatal dopamine deficiency in PD patients. However, as another DRT, it may also activate relatively intact mesocorticolimbic dopaminergic neurons involved in dopaminergic reward pathways [16,17,18,19]. Thus, it is not unexpected that rasagiline may produce, probably in predisposed subjects, addictive or withdrawal symptoms. However, the action of rasagiline on affective symptoms, such as depression and anxiety, has been scarcely explored. This issue is notable if we bear in mind that MAO-B inhibitors have been experimented in the treatment of depression [20]. In this context, it should be remembered as selegiline, another MAO-B inhibitor used in PD, may be effective in treating depression, including refractory geriatric depression [21,22,23]. Noteworthy, a secondary analysis of the DATATOP study revealed that selegiline improved depression compared with a placebo [24]. Depression in PD may be partially related to dopamine deficiency in limbic system pathways [25], and enhancement of dopaminergic neurotransmission via a selective MAO-B inhibitor could therefore interfere in affective pathways related to onset of depression in PD. Moreover, there is evidence that apathy also appears to be mediated by dopaminergic deficiency and improves with DRT [26].

In this emerging scenario, only isolated studies have been directed to the role of rasagiline on depression with contrasting results. Within this context, a previous study showed that rasagiline can improve depressive symptoms in patients with de novo PD [27]. This finding was confirmed by a post hoc exploratory analysis of the ADAGIO study, which showed that depression scores were better in PD patients on rasagiline, suggesting a positive effect for rasagiline on affective non-motor symptoms [28]. On the other hand, these results were not replicated by subsequent research that did not show a significant effect of rasagiline on depressive symptoms in PD patients, although post hoc analyses of the same study indicated some improvements in depression [29].

A possible explanation of the antidepressant effect may be related to the enhanced dopaminergic neurotransmission, since rasagiline inhibits metabolism of dopamine and increases synaptic availability [28]. However, MAO-B inhibitors also affect other monoamines (e.g., phenethylamine) [28]. Furthermore, previous studies showed that rasagiline reduced the progression of physical aspects of fatigue in PD patients, expanding the possible interaction of this drug on different pathways [30,31,32,33].

This report suggests the importance of closely monitoring withdrawal symptoms in PD patients that underwent rasagiline suspension and provides interesting points of reflection that fit into the growing debate on the role of rasagiline in mood disorders. To avoid hypodopaminergic states, PD medication should be adjusted in case of adverse events [11] and PD patients should be always clearly advised by physicians about possible consequences related to the suspension of antiparkinsonian treatment, bearing in mind as well the adverse effects of omitting or delaying antiparkinsonian treatment (especially in a hospital setting) [34].

However, our current knowledge of rasagiline withdrawal syndrome remains incomplete, and future studies are needed both to systematically identify the presence of withdrawal syndrome and to determine if withdrawal of other MAO-B inhibitors could induce similar symptoms.

## Figures and Tables

**Table 1:** Demographic and clinical feature of the three PD patients who developed a withdrawal syndrome after rasagiline suspension.

Case	Age	Sex	Comorbidities	Antiparkinsonian Therapy in Use Other than Rasagiline	Other Medications in Use	Time Elapsing between Withdrawal and Rasagiline Withdrawal Syndrome	Recovery Time after Rasagiline Withdrawal Syndrome Onset	UPDRS-III before Rasagiline Withdrawal	UPDRS-III after Rasagiline Withdrawal Syndrome
1	75	F	Anxiety Constipation Allergic asthma	None	Alprazolam 2 mg/day	10 days	7 months	9	23
2	68	F	None	Rotigotine transdermal patch 4 mg/day	None	7 days	6 months	25	26
3	61	M	None	Pramipexole 1.05 mg/day Levodopa plus carbidopa 100/25 mg for 9 times/day Melevodopa hydrochloride plus carbidopa 100/25 for 2 times/day	None	10 days	9 months	26	28

**Legend:** F, female; M, male, UPDRS-III, Unified Parkinson’s Disease rating scale-Pars III.
